# Quantum-behaved particle swarm optimization based on solitons

**DOI:** 10.1038/s41598-022-18351-0

**Published:** 2022-08-17

**Authors:** Saeed Fallahi, Mohamadreza Taghadosi

**Affiliations:** 1 Department of Mathematics, Salman Farsi University of Kazerun, Kazerun, 73175-457 Iran; 2 Department of Physics, Salman Farsi University of Kazerun, Kazerun, 73175-457 Iran

**Keywords:** Engineering, Mathematics and computing, Physics

## Abstract

This paper introduces a novel variant of the quantum particle swarm optimization algorithm based on the quantum concept of particle-like solitons as the most common solutions of the quantum nonlinear Schrödinger equation. Soliton adaptation in external potentials is one of their most remarkable features which allows them to be stabilized even without a trapping potential, while the potential must be bounded for quantum particles to be localized. So we consider the motion scenario of the present algorithm based on the corresponding probability density function of quantum solitons. To evaluate the efficiency, we examine the proposed algorithm over a set of known benchmark functions, including a selection of test functions with different modalities and dimensions. Moreover, to achieve a more comprehensive conclusion about the performance, we compare it with the results obtained by particle swarm optimization (PSO), standard quantum-behaved particle swarm optimization QPSO, improved sine cosine Algorithm (ISCA), and JAYA optimization algorithm. The numerical experiments reveal that the proposed algorithm is an effective approach to solving optimization problems that provides promising results in terms of better global search capability, high accuracy, and faster convergence rate.

## Introduction

Dealing with complex issues arising from the real world has long been a challenging task. Besides, there are too many intractable optimization problems along with the increasing advances in science and technology that are difficult to solve by traditional methods. Researchers are always looking for efficient algorithms to handle these problems which mostly are non-differentiable and non-continues. Therefore, the use of metaheuristic optimization algorithms is more than ever needed. In recent years a wide number of metaheuristic optimization algorithms are being introduced every day^1,2^. These metaheuristic optimization techniques are generally classified into the following groups, including

- *Evolutionary algorithms (EA)* which are based on natural evolution such as genetic algorithm (GA) and differential evolution^3^.
- *Swarm intelligence algorithms (SI)* that simulate the behavior of animals such as particle swarm optimization (PSO)^4^, salp swarm algorithm^5^, symbiotic organisms search^6^, sine cosine algorithm^7^, and Dolphin echolocation^8^.
- *Classical/modern physics-based (PB) algorithms* which are developed based on physical laws in real-life such as simulated annealing^9^, gravitational and search algorithm^10^.
- *Human-based methods* motivated by human co-operations and human behavior in communities^11^. Such as imperialist competitive algorithm^12^ and teaching-learning-based optimization algorithm^13^.
- *Hybrid algorithms (HA)* which consider different combinations of other algorithms^14^.

The physics-combined version of particle swarm optimization (PSO), which lies in the SI-PB hybrid category, has indicated many appropriate results applicable in different fields of physics and engineering including electromagnetism, gravitation, fluid mechanics, etc. Over the past few decades, the PSO technique as a metaheuristic method has attracted a great deal of interest due to its capability of solving challenging optimization problems^15–17^. Therefore many variants of this algorithm have been proposed and it has achieved great progress in both theory and application. Jain et al.^18^ provided a literature review of PSO during 1995–2017 on the development of PSO, its improvements, variations, and applications.

One such successful PSO variant which is motivated by quantum mechanics is quantum-behaved particle swarm optimization (QPSO). The main idea of emerging quantum probability laws into optimization algorithms was to achieve an oriented and directional search process instead of a completely randomized one. Sun et al.^19^ introduced QPSO in which the particles move through a quantum delta potential well with quantum behavior. They modified the PSO formula and used the average position of all particles’ personal best positions in swarm instead of velocity. The authors also proved that this combination guarantees finding the global optimal solution. QPSO as a probabilistic algorithm has fewer parameters and high capability in solving challenging optimization problems while being simple and easy to implement. Since then, many studies have been done on QPSO. Fang et al.^20^ provided a literature review of QPSO, its improvements, convergence speed, variations, robustness, and applications until 2010. In the course of QPSO evolution, movement behavior, as a key factor in algorithm performance, has attracted a lot of attention in recent years^14,21,22^. In most of them, the motion of a quantum particle under bounded potentials has been considered.

In this paper, as an extension of this approach, we show that it is possible to use localized wave packets in space instead of quantum particles and focus on the swarm optimization techniques inspired by the movement behavior of particle-like solitons which are the analytical solution of the nonlinear Schrödinger (NLS) equation. The NLS equation has been derived in many areas of physics. The mathematical theory of this equation is a broad and very active field of mathematical research. In this equation, the potential term depends on the wave function itself. It is integrable via the Inverse Scattering Transform (IST) and admits multisoliton solutions. It has an infinite number of conserved quantities and possesses many other interesting properties. Solitons, as one of the NLS exact solutions, are ubiquitous in many fields (such as quantum computation, nonlinear optics, plasmas, Bose-Einstein condensate, genome engineering, etc.). They are stable and localized wave packets that behave like particles and maintain their shape while moving at a constant speed. They also remain unchanged during mutual collisions (except possibly for a phase shift) and can reconstruct themselves through the dispersion and nonlinearity of media.

Altogether, according to the mentioned features, it is expected that the new motion scenario which considers the quantum soliton probability density function, allows the particle to have a larger potential space to search and therefore less likely to be stuck in local optima.

The rest of this paper is organized as follows. A brief introduction of PSO, basic principles of QPSO, a review of Solitons, and a description of the proposed algorithm are presented in the “Methodology” section. “Experimental results” section includes the results of optimization and a comparison with some well-known algorithms over 21 benchmark functions. Finally, the conclusions and the future research directions are given in “Summary and conclusion” section.

## Methodology

### Particle swarm optimization (PSO)

Particle swarm optimization belongs to a branch of the SI algorithm that was first intended for simulating social behavior and then developed for constrained and unconstrained problems and also used in discrete and continuous optimization problems. It was first developed by Kennedy and Eberhart in 1995^4^. The main idea of the PSO algorithm is to share the best position of the whole swarm in every generation and then move them toward their own best-known position and the entire swarm’s best-known position in the search space simultaneously. Then, particles are updated according to the following equation:

$$\begin{aligned} V_{i,j}(t+1)= & {} wV_{i,j}(t)+c_{1} r_{1} \left( p_{Best_{i,j}}- X_{i,j}(t) \right) + c_{2} r_{2} (g_{Best_{j}} - X_{i,j}(t)),\\ X_{i,j}(t+1)= & {} X_{i,j}(t)+V_{i,j}(t+1),~\qquad i=1,2, \ldots , M,\qquad j=1,2,\ldots , D, \end{aligned}$$

where $x_{ij}(t)$ and $v_{ij}(t)$ are the value and velocity of $j{\text {th}}$ variable of the $i{\text {th}}$ particle during the $t{\text {th}}$ iteration, respectively. *M* is the size of the population, *D* is the dimension, $p_{Best_{i,j}}$ is the $j{\text {th}}$ variable of the $i{\text {th}}$ best solution so far, $g_{Best_j}$ is the $j{\text {th}}$ variable of the global best particle in the swarm, $c_1$, and $c_2$ are “Personal Learning Coefficient” and “Global Learning Coefficient”, respectively, which are predefined, *w* is a constant, known as “Inertia Weight”, and control the global and local search ability of PSO and $r_1$ and $r_2$ are random numbers in [0, 1]. This process iterates so as to attain the appropriate fitness or reach maximum iterations.

The pseudo-code of the PSO algorithm is illustrated in Algorithm 1.
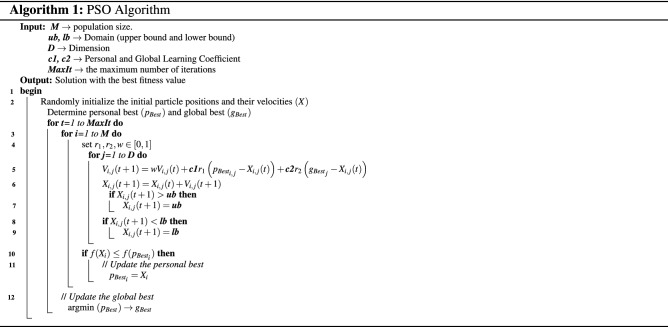


### JAYA algorithm

The JAYA algorithm is a meta-heuristic algorithm recently developed for constrained and unconstrained problems and is also used in discrete and continuous optimization problems. It was first developed by Rao in 2016^23^. In this algorithm, two parameters have to be initialized for any optimization problems: the size of the population, and the maximum number of iterations. Then initial solutions are randomly generated in the feasible region. Later on, the fitness cost of the generated solutions is calculated and the best and the worst solutions in the population are identified. Then each variable of every solution is updated according to the following equation:

1 $$\begin{aligned} X_{i,j}(t+1) = X_{i,j}(t) + r_{1} \left( X_{j,best}- \vert X_{i,j}(t) \vert \right) -r_{2} \left( X_{j,worst}- \vert X_{i,j}(t) \vert \right) , \end{aligned}$$

where $X_{i,j}(t)$ is the value of the $j{\text {th}}$ variable for the $i{\text {th}}$ candidate during the $t{\text {th}}$ iteration, $X_{j,best}$ is the value of the variable *j* for the best candidate and $X_{j,worst}$ is the value of the variable *j* for the worst candidate. $X_{i,j}(t+1)$ is the updated value of $X_{i,j}(t)$ and $r_{1}$ and $r_{2}$ are random numbers in $\left[ 0,1\right]$. If the new solution is in a better condition than the current one, it is replaced by the new one. This procedure is repeated so as to achieve the appropriate fitness or reach maximum iterations.

### Improved sine cosine algorithm

Sine cosine Algorithm (SCA) is a physics-based algorithm and was first developed by Mirjalili et al. in 2016 for solving optimization problems^7^. The main drawback of SCA is having low optimization precision and local minima trapping due to its exploration and exploitation mechanism. In Ref.^24^ the authors proposed a novel strategy to overcome the weakness of the algorithm by substituting a new update mechanism. They proposed a new version of SCA called Improved Sine Cosine algorithm (ISCA). Similar to other meta-heuristic optimization ISCA starts the optimization process by generating the initial solutions randomly in the feasible region. Later on, the fitness cost of the generated solutions is calculated and the best solution in the population is identified. Then each variable of every solution is updated according to the sine and cosine functions as follows:

$$\begin{aligned} X_{i,j}(t+1)=\left\{ \begin{array}{rl} X_{i,j}(t) + r_{1}(t) \sin (r_{2}) \displaystyle \Big \vert r_{3} P_{i,j}(t)- X_{i,j}(t)\Big \vert &{} if \quad r_{4} \geqslant 0.5,\\ X_{i,j}(t) + r_{1}(t) \cos (r_{2}) \displaystyle \Big \vert r_{3} P_{i,j}(t)- X_{i,j}(t)\Big \vert &{} if \quad r_{4} < 0.5, \end{array} \right. \end{aligned}$$

where $X_{i,j}(t)$ is the value of the $j{\text {th}}$ variable for the $i{\text {th}}$ candidate during the $t{\text {th}}$ iteration, $P_{j}(t)$ is the $j{\text {th}}$ variable of the best solution during the $t{\text {th}}$ iteration. $X_{i,j}(t+1)$ is the updated value of $X_{i,j}(t)$ and $r_{1}(t)$ is described as follows:

$$\begin{aligned}r_{1}(t)=a \left( 1- \left( \frac{t}{T} \right) ^\alpha \right) ^\beta ,\end{aligned}$$

in which $\alpha$ and $\beta$, are positive real numbers. $r_{2}$, $r_{3}$, and $r_{4}$ are random numbers in the range $\left[ 0,1\right]$. If the new solution is in a better condition than the current one is replaced by the new one. This procedure is repeated so as to achieve the appropriate fitness or reach maximum iterations.

### Quantum particle swarm optimization (QPSO)

Nature is essentially based on quantum mechanical rules, although the quantum effects are more significant in micro-scale systems. One of the fundamental concepts of quantum mechanics is wave-particle duality, in which all the information about a particle (situation, position, velocity, energy, etc.) is described as a wave function, $\psi (r,t)$, corresponding to a normalized quantum eigenstate. From the quantum point of view, a wave packet, which is a superposition of too many waves, can represent a localized particle in space under a physical potential, but it is broadened to some extent due to the uncertainty principle. According to the Heisenberg uncertainty principle, the exact position of a quantum particle and its velocity cannot be simultaneously determined. That is true for its energy and its quantum state lifetime as well. Therefore, the expectation value of each quantity in quantum mechanics is represented as a probabilistic value, which is determined by the probability density function, $|\psi (r,t)|^2$, which describes the probability of a particle to be found in a given quantum state (a given position, momentum, energy, etc). In the last years, many researchers have tried to introduce quantum concepts through various mathematical frameworks and succeeded in employing them in optimization algorithms. The quantum particle swarm optimization (QPSO) algorithm is inspired by the quantum behavior of nature. The main idea behind the QPSO is to find a proper wave function, associated with a quantum particle in a potential field. To find the optimal solution, QPSO exploits the quantum probability density function to lead particles to the most likely positions (or to the most possible states in a more general sense). Due to the probabilistic nature of quantum mechanics, the correlation between quantum particles, and the mutual influence of their eigenstates, it is expected that the solution lies in the most probable region of the search extent. Although there is not an explicit relation between quantum features and the time complexity, it is expected that the solution lies in the most probable region of the search extent, resulting in better searching performance. The QPSO algorithm, based on some quantum potential fields, such as square well, 1D potential well, the 1D-quantum simple harmonic oscillator, Coulomb-like square root field, Lorentz potential field, and Rosen–Morse has been already used and developed by several authors^14,21,22^. The quantum wave functions utilized in all of the mentioned studies satisfy the usual linear Schrödinger equation,

2 $$\begin{aligned} i\hbar \frac{\partial \psi }{\partial t}+\frac{\hbar ^2}{2m}\frac{\partial ^2\psi }{\partial x^2}-V(x,t)\psi =0. \end{aligned}$$

On the other hand, there are many natural phenomena in physics and engineering, described by nonlinear equations. The Korteweg–de Vries equation, the nonlinear Schrödinger equation, the coupled nonlinear Schrödinger (NLS) equation, and the sine-Gordon equation are some well-known nonlinear equations that have been used extensively in connection with many physical phenomena. They are exactly solvable equations with soliton-like solutions.

In the sequel, we present the probability density function of such problems, especially, quantum solitons with a self-consistent solution to the NLS equation.

### Solitons

**Figure 1:**
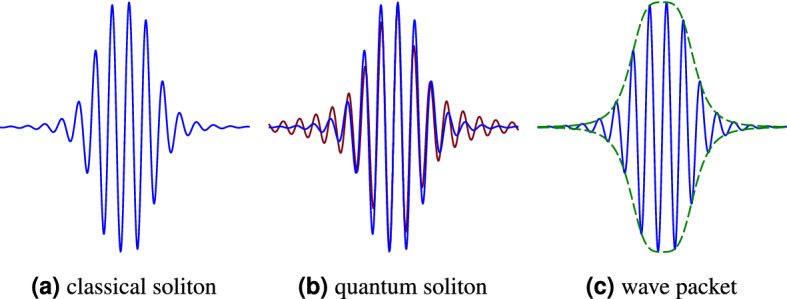
A comparison between soliton, quantum soliton and wave packet.

Solitons or solitary waves are essentially the analytical solutions of physical integrable nonlinear partial differential equations. They are ubiquitous phenomena both in classical and quantum issues with a vast number of applications Classical solitons in physics are non-dispersive pulses, traveling long distances (Fig. 1a). One of the most interesting and unique features of solitons is having neither deformation nor attenuation during propagation in a nonlinear dispersive medium. Taking advantage of the nonlinearity of the medium, solitons can reconstruct themselves, despite dispersion effects. Quantum solitons are indeed the quantum states of classical solutions (Fig. 1b). They are treated as particle-like wave packets with their own coherent eigenstates and energy eigenvalues (Fig. 1c). The quantum solitons are one of the most common solutions of the quantum NLS equation which governs many quantum phenomena in physics and engineering. The usual Schrödinger equation, (Eq. (2)), becomes nonlinear if the potential *V*(*x*, *t*) depends on $\psi$, itself. The general standard dimensionless form of the NLS equation so reads,

3 $$\begin{aligned} i \frac{\partial \psi }{\partial t}+p\frac{\partial ^2\psi }{\partial x^2}+q|\psi |^2\psi =0, \end{aligned}$$

in which $i=\sqrt{-1}$, and *p* and *q* are the coefficients with a definite physical significance. Its basic soliton-like solution is,

4 $$\begin{aligned} \psi (x,t)=\frac{1}{2}\sqrt{\frac{2A}{q}}\frac{\exp (iAt)}{\cosh (\frac{A}{2p}x)}, \end{aligned}$$

where *A* is an arbitrary constant related to the wave packet’s properties (amplitude, width, and frequency). A more general version of the NLS equation is written in terms of the Hamiltonian operator ${\hat{H}}$, as follows:

5 $$\begin{aligned} i \frac{\partial \psi }{\partial t}={\hat{H}}\psi + V\psi +q|\psi |^2\psi . \end{aligned}$$

In this equation, ${\hat{H}}$ corresponds to the related kinetic and potential energies of the system, and is a quadratic function of the momentum operator, ${\hat{p}}=-i\partial /\partial x$ and ${\hat{x}}$, namely, It has a general soliton-like solution as the following form,

6 $$\begin{aligned} \psi (x,t)=\textsf {A}(x,t)F(z)e^{iS(x,t)}, \end{aligned}$$

which describes a traveling soliton with the profile function F(z) in terms of a wave-type argument *z*^25^. The quantum NLS in its general form usually is written as,

7 $$\begin{aligned} i\hbar \frac{\partial \psi }{\partial t}=-\frac{\hbar ^2}{2m}\frac{\partial ^2\psi }{\partial x^2}+2c|\psi |^2\psi , \end{aligned}$$

where, c, as the coupling parameter is a real number. It is positive in the repulsive NLS and negative in the attractive NLS, which arises from the Hamiltonian,

8 $$\begin{aligned} {\hat{H}}=\int ({\psi _x}\psi _x+c\psi ^{*}\psi ^{*}\psi \psi )dx. \end{aligned}$$

The quantum solitons are the solutions of the attractive NLSs. Taking a quick look at the solution of a few problems, one can find the stationary quantum solitons to be as the following standard dimensionless form,

9 $$\begin{aligned} \psi = \alpha \frac{e^{iS}}{\cosh (\beta x)}, \end{aligned}$$

in which, $1/\beta$ denotes the characteristic length defined in the problem. It is worth noting that the wave function, $\psi$, has to be normalized in space, namely,

10 $$\begin{aligned} \int _{-\infty }^{+\infty }|\psi |^2dx=1. \end{aligned}$$

The corresponding probability density function, $|\psi |^2$, is given by,

11 $$\begin{aligned} |\psi |^2=\frac{\alpha ^2}{\cosh ^2(\beta x)}, \end{aligned}$$

plotted in Fig. 2.

**Figure 2:**
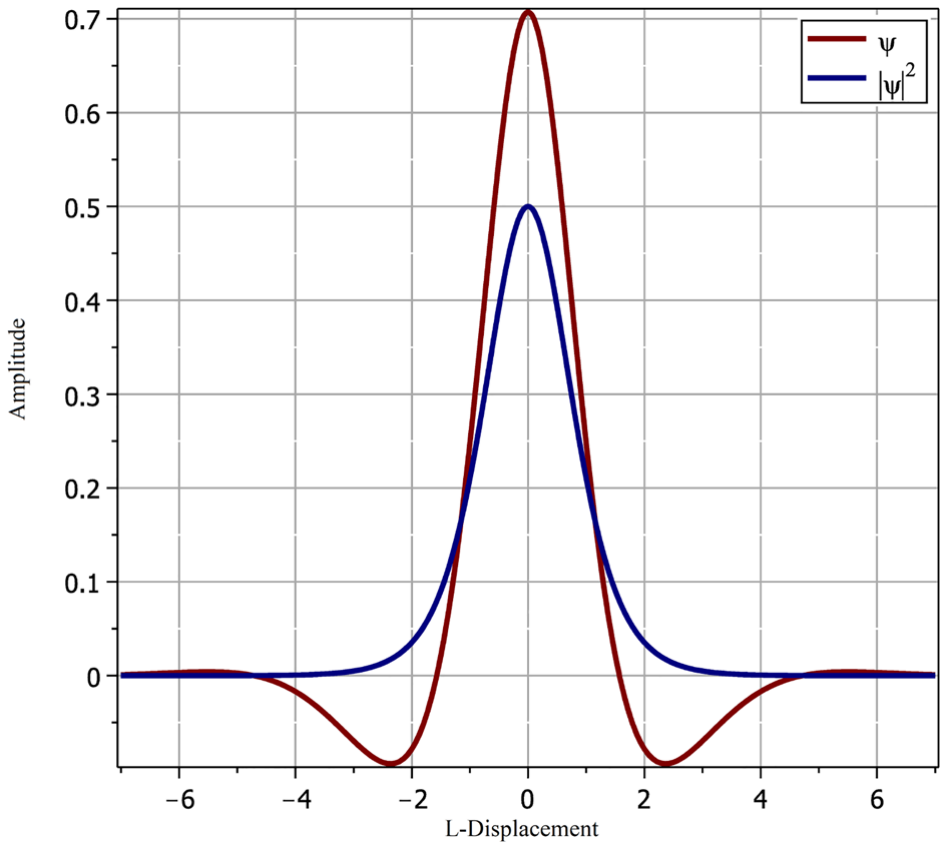
Normalized soliton wave function and the associated probability density function.

As one can see in Supplementary Appendix, although the wave functions can be varied according to the given potential, its general form remains unchanged.

In the next part, we intend to employ the general soliton-solutions of the NLS equation in the QPSO.

### Quantum soliton-inspired optimization algorithms

The main strategy here is exactly the same as the QPSO scenario proposed by many authors^14,19,21,22^, but with an *M* number of particle-like quantum soliton wave packets instead of quantum particles.

In Ref.^26^ the authors have shown that, geometrically, the best position of the particle most likely is located on the linear convex combination of the best local and global position known as local attractor $K_{i}(t)= \left( K_{i,1}(t), K_{i,2}(t), \ldots , K_{i,D}(t) \right)$ defined as follows

12 $$\begin{aligned} K_{i,j}(t)= & {} \lambda ~ p_{Best_{i,j}}(t) + \left( 1- \lambda \right) g_{Best_{j}}(t), \quad i=1,2,\ldots ,M, \quad j=1,2,\ldots ,D, \nonumber \\ \lambda= & {} \frac{c_{1}r_{1}}{c_{1}r_{1}+c_{2}r_{2}}. \end{aligned}$$

According to this trajectory analysis, we have a new movement strategy regarding the best local, $p_{Best_i}(t)$, and global position of the whole swarm, $g_{Best}(t)$. The new position in this model can be updated as follows^14,26^,

13 $$\begin{aligned} x_{i}(t+1)= K_{i}(t)+L ( X_{i}(t), u), \end{aligned}$$

where *L* denotes a displacement function depending on the characteristic length of the problem and a non-uniform distribution function, *F*.

14 $$\begin{aligned} L=\frac{1}{\beta } F. \end{aligned}$$

The characteristic length, representing a physical significance, defined as the absolute difference of average position of all particles’ personal best positions in swarm and the current position, that is

15 $$\begin{aligned} \frac{1}{\beta }= \Big \vert X_{i}(t)-\frac{1}{M}\sum _{i=1}^{M} p_{Best_{i}}(t)\Big \vert . \end{aligned}$$

The distribution function, *F*, which is implicitly related to the probability density function, proposes the most likely position around the local attractor point and is determined as $G^{-1}(u)$, in which *u* is a random number in [0, 1], and G as a random number generator simulated by the probability density function, is assigned to *u*,

16 $$\begin{aligned} \displaystyle G(L)=\frac{\vert \psi (L) \vert ^{2} }{\max (\vert \psi (L) \vert ^{2})}:=u. \end{aligned}$$

By substituting the probability density function of quantum solitons, $\displaystyle |\psi |^2=\alpha ^2/\cosh ^2(\beta L)$, and solving for *L*, we have

$$\begin{aligned}L=\pm \frac{1}{\beta }\cosh ^{-1}\bigg (\frac{1}{\sqrt{u}}\bigg ).\end{aligned}$$

Finally, substituting *L* into Eq. (14), *F*(*u*) is derived as follows,

17 $$\begin{aligned} F(u)=\pm \cosh ^{-1}\bigg (\frac{1}{\sqrt{u}}\bigg ). \end{aligned}$$

Now, by substituting *L* in Eq. (13), the new position can be measured by applying either of the following two equations:

a $$\begin{aligned} X(t+1)= K(t) + \frac{1}{\beta }\cosh ^{-1}\bigg (\frac{1}{\sqrt{u}}\bigg ), \end{aligned}$$

or

b $$\begin{aligned} X(t+1)= K(t) - \frac{1}{\beta }\cosh ^{-1}\bigg (\frac{1}{\sqrt{u}}\bigg ). \end{aligned}$$

According to the quantum mechanical concepts, the quantum state (position) is undetermined until a measurement takes place. So $X(t+1)$ can be updated either by (a) or (b). In the absence of observation, it is indeed a superposition of both. In computation, the random function has the same role as that of the observer in experiments. To do so, let

$$\begin{aligned} X(t+1)=\left\{ \begin{array}{rl} (a) &{} if \quad 0.5 \leqslant \upsilon \leqslant 1,\\ (b) &{} if \quad 0 \leqslant \upsilon < 0.5, \end{array} \right. \end{aligned}$$

where $\displaystyle \upsilon =rand[0,1]$, then, the search radius decreases linearly by multiplying with a factor *w* defined as follows,

18 $$\begin{aligned} \displaystyle w = w_{1} + \frac{ (w_{0} - w_{1}) \,\times\, \left( MaxIt - t \right) }{ MaxIt}, \end{aligned}$$

where $w_0$ and $w_1$ are the initial and final values of *w* respectively. *MaxIt* is the maximum number of iterations and *t* is the current search iteration number. Therefore, Eq. (13) can rewritten as follows,

$$\begin{aligned} X_{i}(t+1)=\left\{ \begin{array}{rl} K_{i}(t) + w \displaystyle \Big \vert \frac{1}{M}\sum _{i=1}^{M} p_{Best_{i}}(t)- X_{i}(t)\Big \vert \cosh ^{-1}\left( \frac{1}{\sqrt{u}}\right) &{} if \quad \upsilon \geqslant 0.5\\ K_{i}(t) - w \displaystyle \Big \vert \frac{1}{M} \sum _{i=1}^{M} p_{Best_{i}}(t)- X_{i}(t)\Big \vert \cosh ^{-1}\left( \frac{1}{\sqrt{u}}\right) &{} if \quad \upsilon <0.5 \end{array}. \right. \end{aligned}$$

The pseudo-code of the quantum soliton-inspired particle swarm optimization (QSPSO) algorithm is illustrated in Algorithm 2.
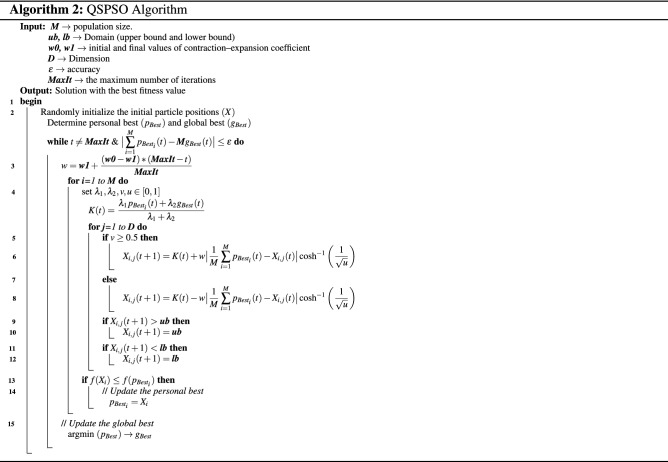


## Experimental results

**Figure 3:**
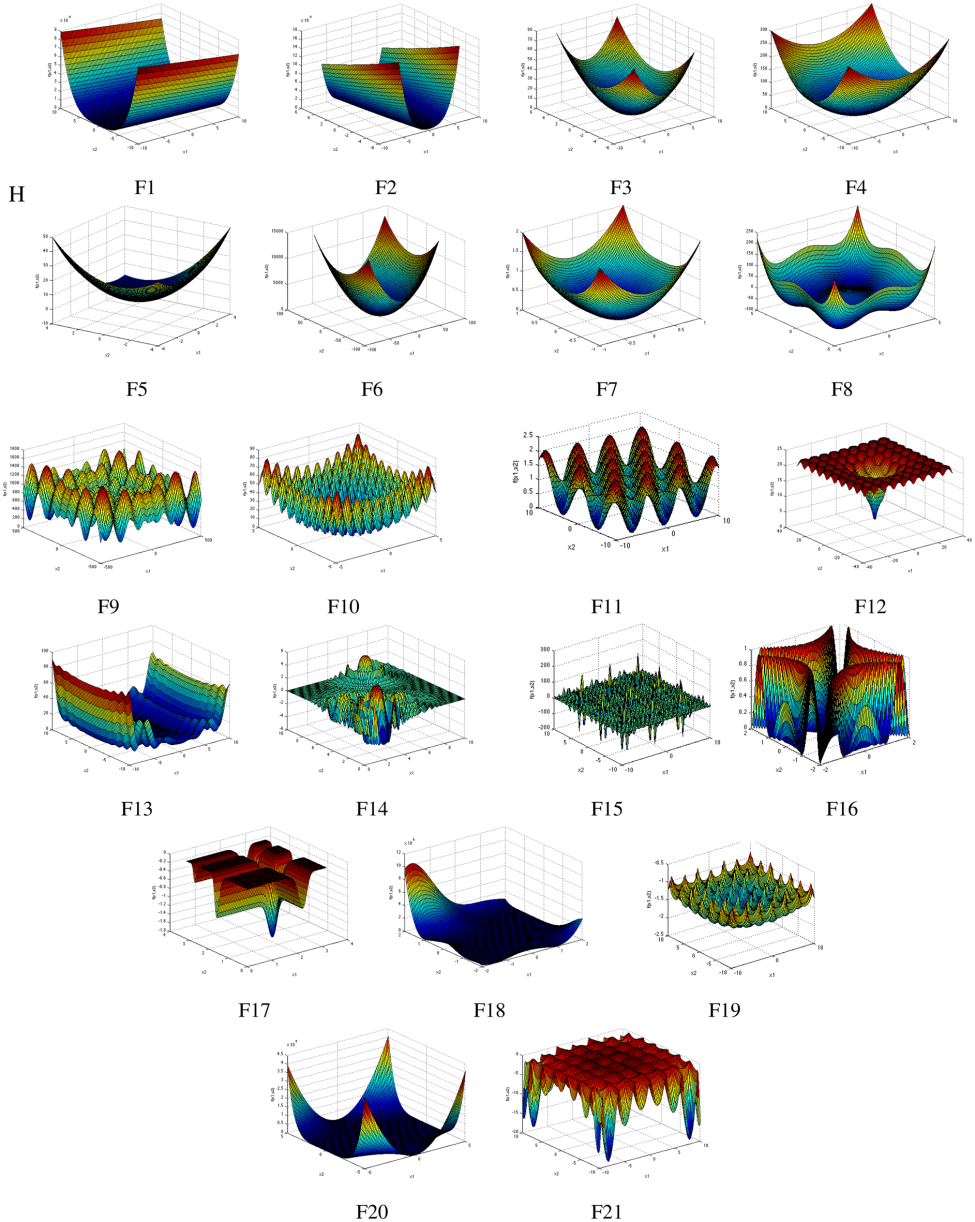
2-D Representation of benchmark functions.

In order to measure and evaluate the performance of the proposed optimization algorithm, it is used to optimize the set of known benchmark functions, including a selection of test functions with different modalities and dimensions (Table 1). These functions are categorized into three classes: unimodal, multimodal, and fixed multimodal functions. A function with a single global optimum is named unimodal. It may or may not be convex and is well for testing the exploitation of algorithms. Multimodal means that the function has more than one local optimum. It is a nonconvex function and is used to test the exploration process of an algorithm and evaluate the ability to escape from any local minimum. Some properties of the used benchmark functions are listed in Table 1 and Fig. 3, where *D* is the dimension of the function, $f_{min}$ is the global optimal value and *Range* indicates the search space. In all experiments, 100 particles are considered as population size for 20 independent runs per function, with the best performance evaluation obtained after 1000 iterations. Computations are performed in MATLAB R2014 on a 2.3 GHz processor with 4 GB of RAM. To evaluate the performance of the algorithm, the following objective criteria are proposed in this paper which target both theexploitation and exploration ability of the algorithm. The first one is accuracy which is defined as the absolute value of the difference between the average (mean) value of the best solutions obtained in the last iteration over multiple runs of an algorithm and the optimal value. Success rate (SR), as the second measure is defined as the percentage of successful runs under a given level of accuracy $(\varepsilon )$ and is defined as the number of successful runs (NSR) divided by the total number of experimental runs and the last measure is computational time performance (Time), which is the average execution time over multiple runs for each optimization algorithm. Moreover, to achieve a more comprehensive conclusion about the performance, we compare it with the results obtained by four other known algorithms on the same optimization problems: particle swarm optimization (PSO), standard quantum-behaved particle swarm optimization (QPSO), improved sine cosine algorithm (ISCA), and JAYA optimization algorithm.

**Table 1:** Benchmark functions^27^.

	Name	Function	D	Range	$f_{min}$
F1	Dixon–Price	$f(x)=(x_1-1)^2+\sum _{i=2}^{D}i\bigg (2x_i^2-x_{i-1}^2\bigg )$	30	$[-\,10, 10]$	0
F2	Rosenbrock	$f(x)=\sum _{i=1}^{D-1}\bigg [100(x_{i+1}-x_i^2)^2+(x_i-1)^2\bigg ]$	30	$[-\,5, 10]$	0
F3	Sphere	$f(x)=\sum _{i=1}^{D}x_i^2$	30	$[-\,5.12, 5.12]$	0
F4	Sum square	$f(x)=\sum _{i=1}^{D}ix_i^2$	30	$[-\,10, 10]$	0
F5	Trid	$f(x)=\sum _{i=1}^{D}(x_i-1)^2-\sum _{i=2}^{D}x_ix_{i-1}$	30	$[-\,900, 900]$	− 4930
F6	Rotated hyper-ellipsoid	$f(x)=\sum _{i=1}^{D}\sum _{j=1}^{i} x_{j}^{2}$	30	$[-\,65.536, 65.536]$	0
F7	Sum of different powers	$f(x)=\sum _{i=1}^{D}\vert x_{i}\vert ^{i+1}$	30	$[-\,1, 1]$	0
F8	Styblinski–Tang	$f(x)=\frac{1}{2}\sum _{i=1}^{D}\bigg (x_i^4-16x_i^2+5x_i\bigg )$	30	$[-\,5, 5]$	− 1175
F9	Schwefel	$f(x)=418.9829D -\sum _{i=1}^{D}x_i \sin (\sqrt{|x_i|})$	30	$[-\,500, 500]$	0
F10	Rastrigin	$f(x)=10D+\sum _{i=1}^{D}\bigg [x_i^2-10\cos (2\pi x_i)\bigg ]$	30	$[-\,5.12, 5.12]$	0
F11	Griewank	$f(x)=\sum _{i=1}^{D}\frac{x_i^2}{4000}-\prod _{i=1}^{D}\cos \bigg (\frac{x_i}{\sqrt{i}}\bigg )+1$	30	$[-\,600, 600]$	0
F12	Ackley	$f(x)=-\,20\exp \bigg (-\,0.2\sqrt{\frac{1}{D}\sum _{i=1}^{D}x_i^2}\bigg )-\exp \bigg (\frac{1}{D}\sum _{i=1}^{D}\cos (2\pi x_i)\bigg )+20+\exp (1)$	30	$[-\,32.768, 32.768]$	0
F13	Levy	$f(x)=\sin ^{2}\left( \pi w_{1} \right) +\sum _{i=1}^{D-1}\left( w_{i}-1\right) ^{2}\left[ 1+10\sin ^{2}\left( \pi w_{i}+1\right) \right] + \left( w_{D}-1\right) ^{2}\left[ 1+ \sin ^{2}\left( 2\pi w_{D} \right) \right]$	30	$[-\,10, 10]$	0
F14	Langermann	$f(x)=\sum _{i=1}^{5}c_{i}\exp \bigg ( -\frac{1}{\pi } \sum _{j=1}^{D}\bigg ( x_{j}-A_{ij} \bigg ) ^2\bigg ) \cos ( \pi \sum _{j=1}^{D}\left( x_{j}-A_{ij} \right) ^2)$	30	[0, 10]	− 1.9341
F15	Shubert	$f(x)=\bigg (\sum _{i=1}^{5}i\cos ((i+1)x_1+i)\bigg )\bigg (\sum _{i=1}^{5}i\cos ((i+1)x_2+i)\bigg )$	5	$[-\,10, 10]$	− 186.7309
F16	Schaffer N.2	$f(x)=0.5+\frac{\sin ^2(x_1^2-x_2^2)-0.5)}{\bigg [1+0.001(x_1^2-x_2^2)\bigg ]^2}$	2	$[-\,100, 100]$	0
F17	Michalewicz	$f(x)=-\sum _{i=1}^{D}\sin (x_i)\sin ^{2m}\bigg (\frac{ix_i^2}{\pi }\bigg )$	5	$[0,\pi ]$	− 4.7033
F18	Goldstein–Price	$f(x)=[1+(x_1+x_2+1)^2(19-14x_1+3x_1^2-14x_2+6x_1x_2+3x_2^2)] \,\times\, [30+(2x_1-3X_2)^2(18-32x_1+12x_1^2+48x_2-36x_1x_2+27x_2^2)]$	2	$[-\,2, 2]$	3
F19	Cross-in-Tray	$f(x)=-\,0.0001\bigg (\bigg |\sin (x_1)\sin (x_2)\exp \bigg (\bigg |100-\frac{\sqrt{x_1^2+x_2^2}}{\pi }\bigg |\bigg )\bigg |+1\bigg )^{0.1}$	2	$[-\,10, 10]$	− 2.0626
F20	Beale	$f(x)=\left( 1.5-x_{1}+x_{1}x_{2} \right) ^{2} + \left( 2.25-x_{1}+x_{1}x_{2}^{2}\right) ^{2} + \left( 2.625-x_{1}+x_{1}x_{2}^{3}\right) ^{2}$	2	$[-\,4.5, 4.5]$	0
F21	Holder Table	$f(x)=- \bigg \vert \sin (x_{1})\cos (x_{2})\exp \left( \bigg \vert 1-\frac{\sqrt{x_{1}^{2}+x_{2}^{2}}}{\pi }\bigg \vert \right) \bigg \vert$	2	$[-\,10, 10]$	− 19.2085

The performance of QSPSO is measured in terms of accuracy, reliability, and computational time. Tables 2, 3 and 4 show the performance results of each algorithm on the solution accuracy, reliability, and computational time for unimodal, multimodal, and fixed-multimodal benchmark functions respectively. In what follows, we compare the algorithms through these criteria.

### Comparisons on the solution accuracy

**Figure 4:**
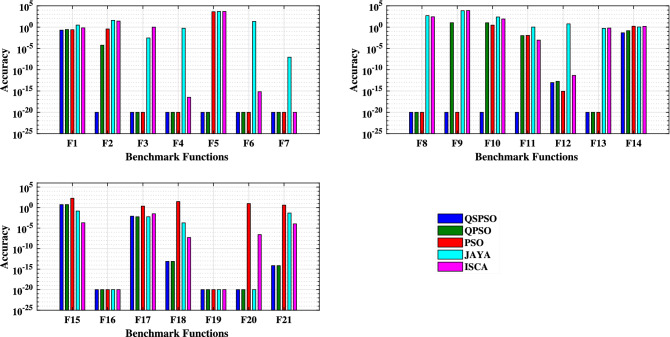
The accuracy comparisons among optimization algorithms. (For each test function, the bar graphs belong to QSPSO, QPSO, PSO, JAYA, and ISCA from left to right, respectively).

Table 2 shows that QSPSO algorithm obtains the most accurate solution in the unimodal function F1, followed by PSO, QPSO, ISCA and JAYA. The QSPSO algorithm also has the best performance in the unimodal functions F2, F3, F4, F6 and F7. Along with QSPSO, PSO and QPSO also perform well and have the best performance in the unimodal functions F3, F4, F6, and F7. For F5, the results of QSPSO and QPSO are the most precise. Analyzing the results obtained from the multimodal benchmark functions, it can be seen from Table 2 that QSPSO obtains the accurate solution and has an excellent performance in almost all of the test instances. It is apparent that PSO also has high accuracy and performs better than other methods on four of the benchmarks (F8, F9, F12, and F13) when searching for function minimum. QPSO also presents a high accuracy for F8, F12, and F13. Evaluating the algorithms for the fixed dimension multimodal benchmark functions shows that QPSO also performs well in most cases, followed by QSPSO. The best results for F15 and F16 are obtained by ISCA and JAYA respectively. ISCA and JAYA also have a good performance in the functions F16 and F19. From Table 4, we can see that among the seven fixed dimension functions, PSO performs poorly with F15, F17, F18, F20, and F21. Figure 4 shows the accuracy results of five algorithms for different benchmark functions.

**Table 2:** Numerical results for unimodal benchmark functions.

Function	D	Algorithm	Mean	Best	Worst	STD	Accuracy	NSR	Time(s)
F1	30	QSPSO	$2.00 \,\times\, 10^{-1}$	$3.16 \,\times\, 10^{-29}$	$6.67 \,\times\, 10^{-1}$	$3.13 \,\times\, 10^{-1}$	$2.00 \,\times\, 10^{-1}$	14	3.25
		QPSO	$2.67 \,\times\, 10^{-1}$	$2.10 \,\times\, 10^{-29}$	$6.67\,\times\, 10^{-1}$	$3.35 \,\times\, 10^{-1}$	$2.67 \,\times\, 10^{-1}$	12	3.50
		PSO	$2.49 \,\times\, 10^{-1}$	$2.49 \,\times\, 10^{-1}$	$2.49\,\times\, 10^{-1}$	$5.70 \,\times\, 10^{-17}$	$2.49 \,\times\, 10^{-1}$	0	4.52
		JAYA	2.99	1.21	6.49	1.50	2.99	0	3.80
		ISCA	$6.67 \,\times\, 10^{-1}$	$6.67 \,\times\, 10^{-1}$	$6.67 \,\times\, 10^{-1}$	$8.47 \,\times\, 10^{-6}$	$6.67 \,\times\, 10^{-1}$	0	4.01
F2	30	QSPSO	0	0	0	0	0	20	1.32
		QPSO	$5.76\,\times\, 10^{-5}$	$3.33 \,\times\, 10^{-9}$	4.19$\,\times\, 10^{-4}$	1.19$\,\times\, 10^{-4}$	5.76$\,\times\, 10^{-5}$	0	4.25
		PSO	$3.69\,\times\, 10^{-1}$	$1.6 5 \,\times\, 10^{-11}$	7.38	1.65	$3.69 \,\times\, 10^{-1}$	0	4.62
		JAYA	4.05$\,\times\, 10^{1}$	2.04$\,\times\, 10^{1}$	1.01$\,\times\, 10^{2}$	2.45$\,\times\, 10^{1}$	4.05$\,\times\, 10^{1}$	0	4.12
		ISCA	2.64$\,\times\, 10^{1}$	2.60$\,\times\, 10^{1}$	2.67$\,\times\, 10^{1}$	1.47$\,\times\, 10^{-1}$	2.64$\,\times\, 10^{1}$	0	4.35
F3	30	QSPSO	0	0	0	0	0	20	2.47
		QPSO	0	0	0	0	0	20	2.65
		PSO	0	0	0	0	0	20	1.21
		JAYA	2.83$\,\times\, 10^{-3}$	1.91$\,\times\, 10^{-3}$	4.28$\,\times\, 10^{-3}$	6.38$\,\times\, 10^{-4}$	283$\,\times\, 10^{-3}$	0	5.07
		ISCA	9.75$\,\times\, 10^{-1}$	7.54$\,\times\, 10^{-1}$	1.25	1.09$\,\times\, 10^{-1}$	9.75$\,\times\, 10^{-1}$	0	4.67
F4	30	QSPSO	0	0	0	0	0	20	2.33
		QPSO	0	0	0	0	0	20	2.25
		PSO	0	0	0	0	0	20	1.36
		JAYA	5.54$\,\times\, 10^{-1}$	3.06$\,\times\, 10^{-1}$	8.68$\,\times\, 10^{-1}$	1.24$\,\times\, 10^{-1}$	5.54$\,\times\, 10^{-1}$	0	4.21
		ISCA	$3.54 \,\times\, 10^{-17}$	$5.19 \,\times\, 10^{-19}$	$2.09 \,\times\, 10^{-16}$	$5.51 \,\times\, 10^{-17}$	$3.54 \,\times\, 10^{-17}$	20	4.65
F5	30	QSPSO	$-4.93\,\times\, 10^{3}$	$-\,4.93\,\times\, 10^{3}$	$-\,4.93\,\times\, 10^{3}$	0	0	20	2.45
		QPSO	$-\,4.93 \,\times\, 10^{3}$	$-\,4.93\,\times\, 10^{3}$	$-\,4.93\,\times\, 10^{3}$	0	0	20	2.68
		PSO	$-\,8.70 \,\times\, 10^{2}$	$-\,8.70\,\times\, 10^{2}$	$-\,8.70\,\times\, 10^{2}$	0	4.06$\,\times\, 10^{3}$	0	4.34
		JAYA	− 3.50$\,\times\, 10^{2}$	− 5.72$\,\times\, 10^{2}$	1.31$\,\times\, 10^{3}$	6.963$\,\times\, 10^{-1}$	4.58$\,\times\, 10^{3}$	0	4.12
		ISCA	− 2.07$\,\times\, 10^{2}$	− 4.58$\,\times\, 10^{2}$	1.04$\,\times\, 10^{3}$	3.11$\,\times\, 10^{2}$	4.72$\,\times\, 10^{3}$	0	4.25
F6	30	QSPSO	0	0	0	0	0	20	1.89
		QPSO	0	0	0	0	0	20	2.12
		PSO	0	0	0	0	0	20	1.66
		JAYA	2.17$\,\times\, 10^{1}$	8.92	3.88$\,\times\, 10^{1}$	8.06	2.17$\,\times\, 10^{1}$	0	4.78
		ISCA	6.83$\,\times\, 10^{-16}$	0	1.90$\,\times\, 10^{-15}$	5.13$\,\times\, 10^{-16}$	6.83$\,\times\, 10^{-16}$	4	4.11
F7	30	QSPSO	0	0	0	0	0	20	1.61
		QPSO	0	0	0	0	0	20	2.47
		PSO	0	0	0	0	0	20	1.77
		JAYA	8.76$\,\times\, 10^{-8}$	1.21$\,\times\, 10^{-9}$	7.31$\,\times\, 10^{-7}$	1.72$\,\times\, 10^{-7}$	8.76$\,\times\, 10^{-8}$	0	3.76
		ISCA	0	0	0	0	0	20	2.71

**Table 3:** Numerical results for multimodal benchmark functions.

Function	D	Algorithm	Mean	Best	Worst	STD	Accuracy	NSR	Time(s)
F8	30	QSPSO	$-\,1.175 \,\times\, 10^{3}$	$-\,1.175 \,\times\, 10^{3}$	$-\,1.175 \,\times\, 10^{3}$	0	0	20	2.30
		QPSO	$-\,1.175 \,\times\, 10^{3}$	$-\,1.175 \,\times\, 10^{3}$	$-\,1.175\,\times\, 10^{3}$	0	0	20	2.62
		PSO	$-\,1.175\,\times\, 10^{3}$	$-\,1.175 \,\times\, 10^{3}$	$-\,1.175\,\times\, 10^{3}$	0	0	20	1.40
		JAYA	$-\,6.85 \,\times\, 10^2$	$-\,7.27 \,\times\, 10^{2}$	$-\,6.437\,\times\, 10^{2}$	$2.30 \,\times\, 10^{1}$	$4.90 \,\times\, 10^{2}$	0	5.23
		ISCA	$-\,9.10 \,\times\, 10^{2}$	$-\,9.50 \,\times\, 10^{2}$	$-\,8.65 \,\times\, 10^{2}$	$2.37 \,\times\, 10^{1}$	$2.65 \,\times\, 10^{2}$	0	5.35
F9	30	QSPSO	$3.82 \,\times\, 10^{-4}$	$3.82 \,\times\, 10^{-4}$	$3.82\,\times\, 10^{-4}$	0	0	20	1.01
		QPSO	$1.09 \,\times\, 10^{1}$	$3.82 \,\times\, 10^{-4}$	2.17$\,\times\, 10^{2}$	4.86$\,\times\, 10^{1}$	1.09$\,\times\, 10^{1}$	19	1.85
		PSO	$3.82 \,\times\, 10^{-4}$	$3.8 2\,\times\, 10^{-4}$	$3.82 \,\times\, 10^{-4}$	$1.38 \,\times\, 10^{-11}$	0	20	1.80
		JAYA	6.95$\,\times\, 10^{3}$	5.09$\,\times\, 10^{3}$	7.50$\,\times\, 10^{3}$	5.25$\,\times\, 10^{2}$	6.95$\,\times\, 10^{3}$	0	4.22
		ISCA	$7.62 \,\times\, 10^{3}$	$6.86 \,\times\, 10^{3}$	$8.04 \,\times\, 10^{3}$	$2.62 \,\times\, 10^{2}$	7.62$\,\times\, 10^{3}$	0	4.38
F10	30	QSPSO	0	0	0	0	0	18	3.02
		QPSO	1.03$\,\times\, 10^{1}$	5.68$\,\times\, 10^{-14}$	2.99$\,\times\, 10^{1}$	1.41$\,\times\, 10^{1}$	1.03$\,\times\, 10^{1}$	11	4.81
		PSO	3.89	1.71$\,\times\, 10^{-13}$	2.90$\,\times\, 10^{1}$	8.90	2.89	12	3.92
		JAYA	2.44$\,\times\, 10^{2}$	2.06$\,\times\, 10^{2}$	2.88$\,\times\, 10^{2}$	$1.95 \,\times\, 10^1$	2.44$\,\times\, 10^{2}$	0	4.65
		ISCA	$8.13\,\times\, 10^{1}$	$2.28 \,\times\, 10^{1}$	$1.20 \,\times\, 10^{2}$	2.70$\,\times\, 10^{1}$	$8.13\,\times\, 10^{1}$	0	4.54
F11	30	QSPSO	0	0	0	0	0	20	0.82
		QPSO	$1.01 \,\times\, 10^{-2}$	0	6.63$\,\times\, 10^{-2}$	$1.63\,\times\, 10^{-2}$	$1.01 \,\times\, 10^{-2}$	12	4.10
		PSO	$1.15 \,\times\, 10^{-2}$	0	$5.63 \,\times\, 10^{-2}$	$1.83\,\times\, 10^{-2}$	$1.15 \,\times\, 10^{-2}$	11	4.31
		JAYA	1.04	1.01	1.06	1.16$\,\times\, 10^{-2}$	1.04	0	4.79
		ISCA	$8.78 \,\times\, 10^{-4}$	0	$1.76 \,\times\, 10^{-2}$	$3.93 \,\times\, 10^{-3}$	$8.78 \,\times\, 10^{-4}$	14	2.93
F12	30	QSPSO	$9.52 \,\times\, 10^{-14}$	3.29$\,\times\, 10^{-14}$	$1.93 \,\times\, 10^{-13}$	$5.03 \,\times\, 10^{-14}$	$9.52 \,\times\, 10^{-14}$	20	2.35
		QPSO	$1.91 \,\times\, 10^{-13}$	$5.06\,\times\, 10^{-14}$	$2.16 \,\times\, 10^{-12}$	4.67$\,\times\, 10^{-13}$	$1.91 \,\times\, 10^{-13}$	20	2.14
		PSO	$8.88 \,\times\, 10^{-16}$	$8.88 \,\times\, 10^{-16}$	$8.88 \,\times\, 10^{-16}$	0	$8.88 \,\times\, 10^{-16}$	20	1.67
		JAYA	5.63	2.38	2.00$\,\times\, 10^{1}$	6.09	5.63	0	4.38
		ISCA	4.48$\,\times\, 10^{-12}$	3.98$\,\times\, 10^{-14}$	1.36$\,\times\, 10^{-11}$	3.56$\,\times\, 10^{-12}$	4.48$\,\times\, 10^{-12}$	20	3.87
F13	30	QSPSO	0	0	0	0	0	20	1.99
		QPSO	0	0	0	0	0	20	1.99
		PSO	0	0	0	0	0	20	1.23
		JAYA	5.18$\,\times\, 10^{-1}$	2.50$\,\times\, 10^{-1}$	1.41	2.77$\,\times\, 10^{-1}$	5.18$\,\times\, 10^{-1}$	0	4.11
		ISCA	5.98$\,\times\, 10^{-1}$	5.07$\,\times\, 10^{-1}$	7.02$\,\times\, 10^{-1}$	6.48$\,\times\, 10^{-2}$	5.98$\,\times\, 10^{-1}$	0	4.49
F14	30	QSPSO	− 1.89	− 1.93	− 1.02	2.04$\,\times\, 10^{-1}$	4.55$\,\times\, 10^{-2}$	19	2.57
		QPSO	− 1.79	− 1.93	− 1.02	5.94$\,\times\, 10^{-1}$	1.42$\,\times\, 10^{-1}$	15	3.06
		PSO	− 2.86$\,\times\, 10^{-1}$	− 2.86$\,\times\, 10^{-1}$	− 2.86$\,\times\, 10^{-1}$	7.73$\,\times\, 10^{-13}$	1.65	0	2.58
		JAYA	− 8.44$\,\times\, 10^{-1}$	− 1.93	− 2.86$\,\times\, 10^{-1}$	5.88$\,\times\, 10^{-1}$	1.09	0	3.73
		ISCA	− 3.95$\,\times\, 10^{-1}$	− 1.02	− 1.08$\,\times\, 10^{-3}$	4.70$\,\times\, 10^{-1}$	1.54	0	4.06

**Table 4:** Numerical results for fixed dimension multimodal benchmark functions.

Function	D	Algorithm	Mean	Best	Worst	STD	Accuracy	NSR	Time(s)
F15	5	QSPSO	− 1.81$\,\times\, 10^{2}$	− 1.87$\,\times\, 10^{2}$	7.94$\,\times\, 10^{1}$	2.40$\,\times\, 10^{1}$	5.37	16	2.97
		QPSO	− 1.81$\,\times\, 10^{2}$	− 1.87$\,\times\, 10^{2}$	7.94$\,\times\, 10^{1}$	2.40$\,\times\, 10^{1}$	5.37	14	3.30
		PSO	2.71$\,\times\, 10^{-29}$	1.97$\,\times\, 10^{-31}$	3.65$\,\times\, 10^{-28}$	8.04$\,\times\, 10^{-29}$	1.87$\,\times\, 10^{2}$	0	4.62
		JAYA	− 1.87$\,\times\, 10^{2}$	− 1.87$\,\times\, 10^{2}$	− 1.86$\,\times\, 10^{2}$	2.17$\,\times\, 10^{-1}$	1.47$\,\times\, 10^{-1}$	15	2.50
		ISCA	− 1.87$\,\times\, 10^{2}$	− 1.87$\,\times\, 10^{2}$	− 1.87$\,\times\, 10^{2}$	2.00$\,\times\, 10^{-4}$	2.01$\,\times\, 10^{-4}$	20	1.74
F16	2	QSPSO	0	0	0	0	0	20	1.18
		QPSO	0	0	0	0	0	20	0.92
		PSO	0	0	0	0	0	20	0.87
		JAYA	0	0	0	0	0	20	2.84
		ISCA	0	0	0	0	0	20	1.15
F17	5	QSPSO	− 4.70	− 4.71	− 4.67	1.63$\,\times\, 10^{-2}$	7.92$\,\times\, 10^{-3}$	17	2.10
		QPSO	− 4.70	− 4.71	− 4.67	1.45$\,\times\, 10^{-2}$	$5.94 \,\times\, 10^{-3}$	18	2.13
		PSO	− 2.46	− 2.46	− 2.46	2.28$\,\times\, 10^{-16}$	2.25	0	4.82
		JAYA	− 4.70	− 4.71	− 4.67	1.45$\,\times\, 10^{-2}$	$5.94 \,\times\, 10^{-3}$	18	2.83
		ISCA	− 4.68	− 4.71	− 4.66	$1.15 \,\times\, 10^{-2}$	3.10$\,\times\, 10^{-2}$	16	3.01
F18	2	QSPSO	3.00	3.00	3.00	4.23$\,\times\, 10^{-16}$	7.82$\,\times\, 10^{-14}$	20	0.81
		QPSO	3.00	3.00	3.00	1.81$\,\times\, 10^{-16}$	$7.82\,\times\, 10^{-14}$	20	0.96
		PSO	$3.27\,\times\, 10^{1}$	$3.27\,\times\, 10^{1}$	$3.27\,\times\, 10^{1}$	0	$2.97 \,\times\, 10^{1}$	0	4.42
		JAYA	3.00	3.00	3.00	1.59$\,\times\, 10^{-4}$	1.93$\,\times\, 10^{-4}$	20	1.57
		ISCA	3.00	3.00	3.00	$4.78 \,\times\, 10^{-8}$	$5.24 \,\times\, 10^{-8}$	20	1.39
F19	2	QSPSO	− 2.06	− 2.06	− 2.06	0	0	20	1.28
		QPSO	− 2.06	− 2.06	− 2.06	0	0	20	1.25
		PSO	− 2.06	− 2.06	− 2.06	0	0	20	0.92
		JAYA	− 2.06	− 2.06	− 2.06	0	0	20	1.60
		ISCA	− 2.06	− 2.06	− 2.06	0	0	20	1.47
F20	30	QSPSO	0	0	0	0	0	20	1.41
		QPSO	0	0	0	0	0	20	1.28
		PSO	9.71	9.71	9.71	3.65$\,\times\, 10^{-15}$	9.71	0	2.20
		JAYA	0	0	0	0	0	20	1.86
		ISCA	2.70$\,\times\, 10^{-7}$	7.50$\,\times\, 10^{-9}$	1.10$\,\times\, 10^{-6}$	3.43$\,\times\, 10^{-7}$	2.70$\,\times\, 10^{-7}$	0	2.38
F21	30	QSPSO	− 1.92$\,\times\, 10^{1}$	− 1.92$\,\times\, 10^{1}$	− 1.92$\,\times\, 10^{1}$	6.87$\,\times\, 10^{-15}$	7.11$\,\times\, 10^{-15}$	17	1.36
		QPSO	− 1.92$\,\times\, 10^{1}$	− 1.92$\,\times\, 10^{1}$	− 1.92$\,\times\, 10^{1}$	6.10$\,\times\, 10 \,\times\, 10^{-15}$	7.11$\,\times\, 10^{-15}$	19	1.33
		PSO	− 1.51$\,\times\, 10^{1}$	− 1.51$\,\times\, 10^{1}$	− 1.51$\,\times\, 10^{1}$	3.65$\,\times\, 10^{-15}$	4.07	0	2.82
		JAYA	− 1.92$\,\times\, 10^{1}$	− 1.92$\,\times\, 10^{1}$	− 1.88$\,\times\, 10^{1}$	1.10$\,\times\, 10^{-1}$	4.84$\,\times\, 10^{-2}$	7	1.91
		ISCA	− 1.92$\,\times\, 10^{1}$	− 1.92$\,\times\, 10^{1}$	− 1.92$\,\times\, 10^{1}$	9.56$\,\times\, 10^{-5}$	1.08$\,\times\, 10^{-4}$	6	1.62

### Comparisons of the convergence speed and reliability

**Figure 5:**
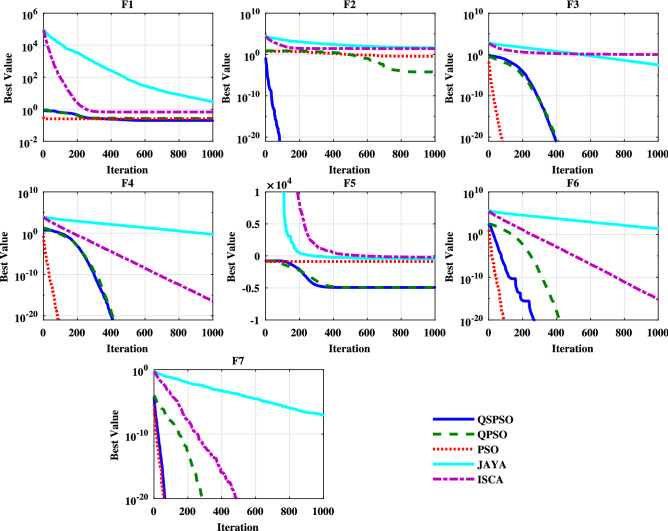
Convergence curves of the unimodal test functions.

**Figure 6:**
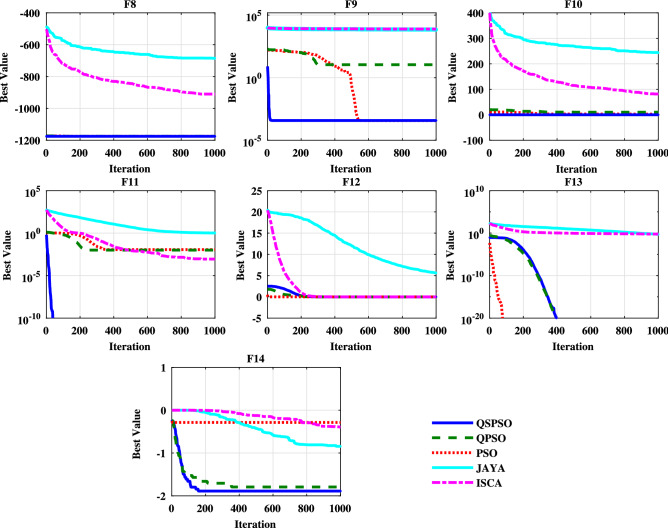
Convergence curves of the multimodal test functions.

**Figure 7:**
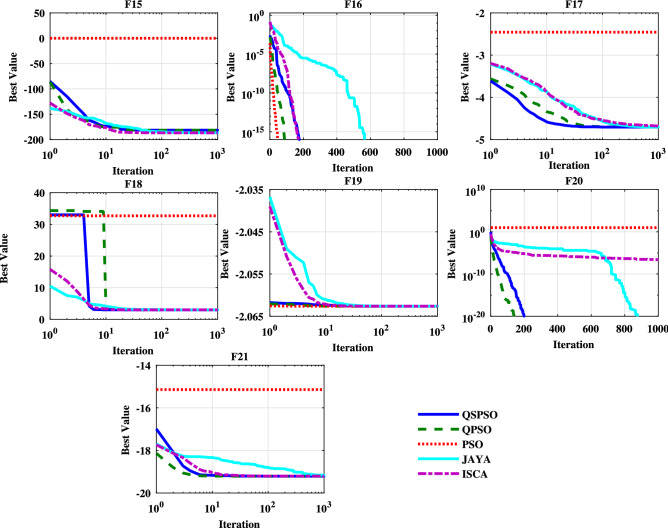
Convergence curves of the fixed-multimodal test functions.

Reliability or the success rate is computed as the percentage of experimental runs reaching optimal solutions. Table 5 shows the percentage of trials achieving acceptable solutions. It shows that compared to other algorithms, the QSPSO algorithm achieves considerably better results and can reach acceptable solutions in more experiments over all the benchmark functions. Moreover, Figs. 5, 6 and 7 present the converging curves for the unimodal, multimodal, and fixed multimodal benchmark functions respectively. These graphs illustrate the mean of the best fitness value according to the $i{\text {th}}$ iteration and help us to evaluate the convergence speed of the algorithm. From Fig. 5, it can be obtained that for the unimodal functions, OSPSO and OPSO algorithms behave almost the same for F1, F3, F4, and F5. In F2, F6, and F7, compare with QPSO, QSPSO converges faster and can reach the optimal point during the early stage of optimization. For F3, F4, F6, and F7 PSO performs well and sharply drops off and requires less iteration than QSPSO and QPSO. In this case, JAYA and ISCA show poor performance. For the multimodal functions, Figure 6 shows that QSPSO almost performs better followed by PSO than the other algorithms. JAYA shows poor performance for multimodal functions. From Fig. 7, it can be realized that for each algorithm, there is a better performance depending on the function in the case of the fixed multimodal functions. All algorithms except PSO can reach the near-optimal point.

**Table 5:** Reliability of and comparisons among algorithms ($\%$).

Functions	QSPSO	QPSO	PSO	JAYA	ISCA
F1	70	60	0	0	0
F2	100	0	0	0	0
F3	100	100	100	0	0
F4	100	100	100	0	20
F5	100	100	0	0	0
F6	100	100	100	0	20
F7	100	100	100	0	100
F8	100	100	100	0	0
F9	100	95	100	0	0
F10	90	55	60	0	0
F11	100	60	55	0	70
F12	100	100	100	0	100
F13	100	100	100	0	0
F14	95	75	0	0	0
F15	80	70	0	75	100
F16	100	100	100	100	100
F17	85	90	0	90	80
F18	100	100	0	100	100
F19	100	100	100	100	100
F20	100	100	0	100	0
F21	85	95	0	35	30

### Comparisons on the computational time

**Figure 8:**
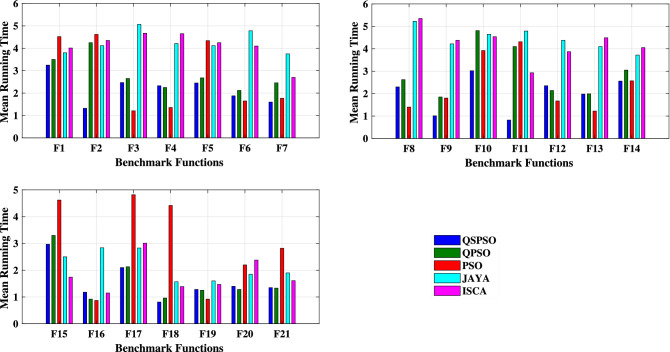
The average running time for optimization algorithms on the test functions. (For each test function, the bar graphs belong to QSPSO, QPSO, PSO, JAYA, and ISCA from left to right, respectively).

The comparison of the average running time of the proposed algorithm with other well-known optimization algorithms is presented in Tables 2, 3 and 4. Figure 8 also shows the average running time for each optimization algorithm. As can be seen in most cases, the proposed algorithm is able to reach the optimum solution in a shorter time compared to the other mentioned algorithms.

## Summary and conclusion

Quantum-behaved swarm optimization, as one of the most efficient variants of PSO, is a very effective and powerful technique that has been widely used in solving optimization problems. The QPSO in the common versions is based on the quantum behavior of particles under a limited potential which is generally described by the usual quantum schrödinger equation. By providing a few examples, we showed that solitons are the most common solutions to the quantum nonlinear Schrödinger equation. Solitons can be localized and stable even without a trapping potential and they can also reproduce and rearrange themselves in nonlinear conditions. So, in this study, we considered the quantum concept of particle-like solitons and introduced a new version of QPSO mentioned here as QSPSO. Implementing the algorithm on different types of benchmark functions and comparison to some state-of-art meta-heuristic algorithms, such as PSO, standard QPSO, ISCA, and JAYA, we showed that if the motion scenario of the algorithm is designed based on the corresponding probability density function of quantum solitons, the global search capability of the algorithm will be improved. According to numerical experiments, it is deduced that the new motion scenario which considers the quantum soliton probability density function, allows the particle to have a larger potential space to search and therefore less likely to be stuck in local optima. The results show that QSPSO has a good overall performance in terms of accuracy, reliability, and computational time. Regarding the obtained results, considering the other types of solitons and the related probability distributions as a candidate for the motion scenario would be interesting for future works.

## Supplementary Information

Supplementary Information.

## Data Availability

The datasets used and/or analysed during this study are included in this published article.
