# Supplementary material for: Quantum-behaved particle swarm optimization based on solitons
Source: Sci Rep. 2022 Aug 17;12:13977. doi: 10.1038/s41598-022-18351-0 (PMC9385677; doi:10.1038/s41598-022-18351-0)
Supplement: Supplementary file 1 — Supplementary Information. [file 41598_2022_18351_MOESM1_ESM.pdf]

Appendix A. On the Solitons and their Applications

Some examples of solitons in various fields are presented as follows.

(i) Optical telecommunication. Optical telecommunication over intercontinental distances is based on the propagation of soliton optical pulses through a single-mode nonlinear optical fiber. The optical soliton can be approximately described by the classical time-dependent wave function:

$$\psi(X,T) = \sqrt{\frac{\bar{N}}{2}}\frac{e^{i(\theta_0+T/2)}}{\cosh(X)},$$

where $\bar{N}$, X, T and θ_0 are the mean photon number, dimensionless position, dimensionless time, and phase constant, respectively. The quantum solitons in optical fibers, however, have slightly different behavior. They are superpositions of too many solitons with different photon numbers, different phases, and different eigenstates. In this case, the classical fields are substituted by operators [1]. The corresponding NLS equation in terms of the quantum density amplitude operator, $\hat{\psi}$, is given by a dimensionless attractive NLS equation:

$$i\frac{\partial}{\partial T}\hat{\psi}(X,T) = (-\frac{1}{2}\frac{\partial^2}{\partial X^2} + \frac{2}{\bar{N}}\hat{\psi}^{\dagger}\hat{\psi})\hat{\psi}(X,T).$$

The corresponding matrix elements based on the coherent quantum soliton states, $|n,P>$, are given by,

$$<n,P'|\hat{\psi}(x)|n+1,p>= \frac{\pi}{\sqrt{|c|}}\frac{\exp\left(i(P-P')x\right)}{\cosh(\frac{\pi}{\sqrt{|c|}}(\frac{P'}{n}-\frac{P}{n+1}))},$$

where n and P are quantum number and momentum, respectively.
Using a Fourier transform to coordinate space for large enough values of n, this equation can be written in the same form as the classical soliton wave [1, 2],

$$\psi(x,t) = \alpha\frac{e^{iS(x,t)}}{\cosh(2\eta(x-x_0-vt))},$$

in which $\alpha = 2\eta/\sqrt{|c|}$ is the normalization factor, $S(x,t) = -4i(\xi^2-\eta^2)t - 2i\xi x$, and $\zeta = \xi + i\eta$ is the corresponding eigenvalue.

(ii) Quantum information. Quantum information processing or quantum computation relies on qubits. They can be obtained from modern techniques, one of which is based on photon trapping by optical solitons [3].
The related quantum states in this case, can be expressed by:

$$\psi_i(x,z) = \alpha\frac{e^{iS(x,t)}}{\cosh(a(x-x_i))}, \qquad S(x,t) = 2i\mu(t)(x-\bar{x}_i) + i\delta_i,$$

as the solution of the following NLS equation [4],

$$i\partial_z\psi + \frac{1}{2}\partial_x^2\psi + |\psi|^2\psi = 0.$$

Quantum signatures and secure information transfer are also the other novel applications proposed for quantum solitons [4, 5].

(iii) DNA deformation. The solitons can be found even in the brain synapses and in genome engineering [6, 7]. Englander *et al.* studied the twist deformation propagating along adjacent DNA base pairs and demonstrated the possibility of thermally induced soliton excitations within a DNA double helix chain [8]. By considering the self-organized electron states (SES), Lakhno showed that the superexchange electron transfer (ET) mechanism in DNA also leads to a soliton-like excitation [9]. In his simple model of an elastic DNA thread, (Figure 1), the corresponding wave function of donor/acceptor, ψ_D/ψ_A, localized in a rectangular potential well is as the form

$$\psi_{D/A}(x) = \sqrt{(\frac{2}{d})}\sin(\frac{\pi}{d}x), \qquad \frac{2}{3}R-d \leq |x| \leq \frac{2}{3}R.$$

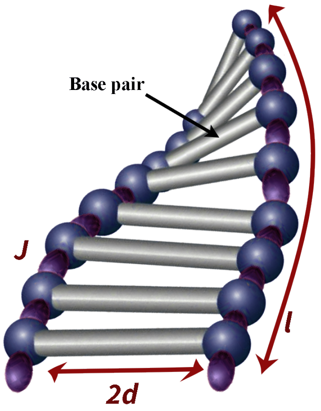

Figure 1. A simple model for a short fragment of DNA with length l

R is the molecular size and l is the length of DNA. According to [10], the SES and also soliton solutions are valid for DNAs of length larger than the minimal length, $l_{min} = 2R_{min} \simeq 42$Å. The electron eigenstates depend on the density of DNA elasticity energy, and its interaction with deformation. The electron wave function, $\psi(x,t)$, is governed by:

$$i\hbar\frac{\partial\psi(x,t)}{\partial t} + \frac{\hbar^2}{2m}\frac{\partial^2\psi(x,t)}{\partial x^2} + G\psi(x,t)\frac{\partial}{\partial x}u(x,t) = 0, \qquad |x| \leq R,$$
$$i\hbar\frac{\partial\psi(x,t)}{\partial t} + \frac{\hbar^2}{2m}\frac{\partial^2\psi(x,t)}{\partial x^2} = 0, \qquad |x| > R,$$

in which, G is the deformation potential and $u(x,t)$ is the displacement at each point of the DNA thread. The soliton-like solution for a long strand of DNA is given by

$$\psi(x,t) = \frac{1}{\sqrt{2r}}\frac{exp(\frac{i}{\hbar}(\frac{1}{2}mv^2 - w)t)exp(\frac{i}{\hbar}mv(x-vt))}{\cosh(\frac{1}{r}(x-vt))},$$

which in stationary form reads

$$\psi(x) = \frac{1}{\sqrt{2r}}\frac{exp(\frac{i}{\hbar}mvx)}{\cosh(\frac{x}{r})},$$

where, $r = \frac{2\hbar^2}{m}\frac{k}{G^2}(l - \frac{v^2}{c^2})$ is the characteristic size of soliton, $w = -\hbar^2/2mr^2$ is the electron energy, k is the elasticity constant, $c = \sqrt{k/\rho}$ is the sound velocity, and ρ is the density of the thread. For the short thread of DNA, there is a discrete number of solutions, one of them in the stationary dimensionless form is given by:

$$\psi(x) = \frac{\sqrt{2k|w|/G^2\hbar^2}}{\cosh(\sqrt{2m|w|/\hbar^2 x})}.$$

References

1. Drummond, P., Shelby, R., Friberg, S. & Yamamoto, Y. Quantum solitons in optical fibres. *Nature* **365**, 307–313 (1993).

2. Bullough, R. & Wadati, M. Optical solitons and quantum solitons. *J. Opt. B: Quantum Semiclassical Opt.* **6**, S205 (2004).

3. Steiglitz, K. Soliton-guided quantum information processing. In *Advances in Unconventional Computing*, 297–307 (Springer, 2017).

4. Silva, N. A., Ferreira, T. D. & Guerreiro, A. Reservoir computing with solitons. *New J. Phys.* **23**, 023013 (2021).

5. Abram, I. Quantum solitons. *Phys. world* **12**, 21 (1999).

6. Tabi, C., Mohamadou, A. & Kofané, T. *Solitons in DNA and Biological Implications* (Scholar's Press, 2014).

7. Cuenda, S., Sánchez, A. & Quintero, N. R. Does the dynamics of sine–gordon solitons predict active regions of dna? *Phys. D: Nonlinear Phenom.* **223**, 214–221, DOI: https://doi.org/10.1016/j.physd.2006.09.005 (2006).

8. Englander, S., Kallenbach, N., Heeger, A., Krumhansl, J. & Litwin, S. Nature of the open state in long polynucleotide double helices: possibility of soliton excitations. *Proc. Natl. Acad. Sci.* **77**, 7222–7226 (1980).

9. Lakhno, V. Soliton-like solutions and electron transfer in dna. *J. Biol. Phys.* **26**, 133–147 (2000).

10. Korshunova, L. V., A.N. & Shnol, E. Solutions of nonlinear selfconsistent problem for electron in cluster placed in strong magnetic field. *Proc. Natl. Acad. Sci.* **44**, 399—402 (1999).
